# A flow-injection mass spectrometry fingerprinting scaffold for feature selection and quantitation of *Cordyceps* and *Ganoderma* extracts in beverage: a predictive artificial neural network modelling strategy

**DOI:** 10.1186/2191-0855-2-43

**Published:** 2012-08-13

**Authors:** Chee Wei Lim, Siew Hoon Tai, Sheot Harn Chan

**Affiliations:** 1 Food Safety Laboratory, Applied Sciences Group, Health Sciences Authority, 11 Outram Road, Singapore, 169078, Singapore; 2 AB SCIEX (Distribution), 10 Biopolis Road, #03-06, Chromos, 138670, Singapore

**Keywords:** *Cordyceps sinensis*, *Ganoderma lucidum*, MS, Fingerprinting, ANN, Quantitation

## Abstract

Flow-injection mass spectrometry (FI/MS) represents a powerful analytical tool for the quality assessment of herbal formula in dietary supplements. In this study, we described a scaffold (proof-of-concept) adapted from spectroscopy to quantify *Cordyceps sinensis* and *Ganoderma lucidum* in a popular *Cordyceps sinensis* /*Ganoderma lucidum* -enriched health beverage by utilizing flow-injection/mass spectrometry/artificial neural network (FI/MS/ANN) model fingerprinting method with feature selection capability. Equal proportion of 0.1% formic acid and methanol (v/v) were used to convert extracts of *Cordyceps sinensis* and *Ganoderma lucidum* into their respective ions under positive MS polarity condition. No chromatographic separation was performed. The principal *m*/*z* values of *Cordyceps sinensis* and *Ganoderma lucidum* were identified as: 104.2, 116.2, 120.2, 175.2, 236.3, 248.3, 266.3, 366.6 and 498.6; 439.7, 469.7, 511.7, 551.6, 623.6, 637.7 and 653.6, respectively. ANN models representing *Cordyceps sinensis* and *Ganoderma lucidum* were individually trained and validated using three independent sets of matrix-free and matrix-matched calibration curves at concentration levels of 2, 20, 50, 100, 200 and 400 μg mL^-1^. Five repeat analyses provided a total of 180 spectra for herbal extracts of *Cordyceps sinensis* and *Ganoderma lucidum*. Root-mean-square-deviation (RMSE) were highly satisfactory at <4% for both training and validation models. Correlation coefficient (*r*^2^) values of between 0.9994 and 0.9997 were reported. Matrix blanks comprised of complex mixture of Lingzhi fermentation solution and collagen. Recovery assessment was performed over two days using six sets of matrix blank (*n* = 6) spiked at three concentration levels of approximately 83, 166 and 333 mg kg^-1^. Extraction using acetonitrile provided good overall recovery range of 92-118%. A quantitation limit of 0.2 mg L^-1^ was reported for both *Cordyceps sinensis* and *Ganoderma lucidum*. Intra-day and inter-day RMSE values of 7% or better were achieved. Application of the scaffold in a high-throughput routine environment would imply a significant reduction in effort and time, since the option of having a model driven analytical solution is now available.

## Introduction

Flow injection mass spectrometry (FI/MS) represents a proven analytical tool for the qualitative and quantitative analyses of chemical residues in complex matrices (Nanita et al. [2011]; Nanita [2011]; Kristiansen et al. [1994]; Nanita et al. [2009]). It has also been successfully applied for qualitative high-throughput metabolite fingerprinting (Enot et al. [2008]; Beckmann et al. [2008]) and screening (Roddy et al. [2007]) in drugs discovery. Owing to the complex compositional variation of food matrices, traditional FI/MS strategy involved the application of a chromatographic step to separate the targeted analyte of interest before detection, thereby generating quantitative or semi quantitative information on individual analytes. Such targeted analyte profiling strategy demands strict control over the chromatographic process to obtain reproducibility (Lisec et al. [2006]). With extended use, it becomes inevitable that chromatographic column undergoes gradual deterioration, thereby contributing toward significant changes in data characteristics such as peak-shape asymmetry after performing high-volume profiling experiments. For a high-throughput routine laboratory with broad analytical base, the cost of ownership (COO) associated with the sustenance of chromatographic accessories therefore presents a steep challenge, amongst others.

An alternative approach to capture information relating to chemical residues in complex matrices is to develop a spectrometric fingerprinting strategy without performing chromatographic separation (Enot et al. [2006]; Ward et al. [2003]), assisted by an artificial neural network model (ANN). Indeed, the concept of applying ANN as an analytical tool in analytics is not new (Marini [2009]). ANN has been successfully applied as a classifying tool (Debska and Guzowska-Świder [2011]; Galdón et al. [2010]) in food categorization, as well as an analytical tool for the determination of potential endocrine disruptors (Boto et al. [2009]) and oligosaccharides (Onofrejová et al. [2007]) in food. In this study, we present a proof-of-concept that the application of a FI/MS tandem ANN approach represents a viable analytical platform to address emerging concerns associated with technical difficulties encountered when performing quality assessment of complex herbal formula, namely *Cordyceps sinensis***(C)** and *Ganoderma lucidum***(G)** in beverage. Owing to the complex chemical fingerprint of **(C)** and **(G)** and whose composition varies with species, cultivation practices and harvests (Yang et al. [2009]), conventional analytical strategy of using targeted analysis aided by a suitably chosen chromatographic separation technique presents a steep challenge to analysts worldwide. For this reason, it is the objective of this paper to equip analysts with an alternative tool presented in the form of a scaffold for the determination of herbal extracts of **(C)** and **(G)** in beverage, even when exact information pertaining to the total chemical fingerprints of both herbal extracts is not readily available. Validation/ANN model optimization was performed by applying criteria adapted from those detailed by Mol and colleagues for simultaneous determination of chemical residues in feed and food matrices (Mol et al. [2008]), and Belgrano and colleagues for the application of ANN to primary production time-series data (Belgrano et al. [2001]), respectively.

## Materials and methods

### Instrumentation

The flow-injection mass spectrometery (FI/MS) system consisted of an Agilent model 1290 infinity LC (Palo Alto, CA, USA) coupled to a QTrap 5500 MS instrument (AB SCIEX, Foster city, CA, USA) mass spectrometer. The LC system comprised four solvent reservoirs, a built-in degasser, two binary pumps and a refrigerated autosampler. A 0.3 μm pre-column inline filter (Agilent Technologies, Palo Alto, CA, USA) was used to minimize potential contamination to the MS system; the pre-column inline filter did not provide meaningful separation. Mobile phases consisted of 0.1% formic acid (A) and MeOH (B) with isocratic elution at 50:50 (v/v) at a flow rate of 0.4 mL min^-1^ for 1 min.

Electrospray ionization (ESI) was performed in positive ion mode from *m*/*z* 100 to 700 using the linear ion trap (LIT) operating under enhanced MS (EMS) mode. Under EMS mode, a full MS scan was performed, during which ions were trapped for a certain period before being detected in the spectrometer. Ion trap fill time was set to dynamic to address *m*/*z* shifts due to space-charging effects. The following MS conditions were used: source temperature and gas flows were set to 500°C and 45 psi, respectively; declustering potential (DP) was set to 100 V; electrospray voltage was set to 5 kV; curtain gas was set to 30. A scan speed of 10 kDa s^-1^ at unit resolution (0.7 Da) was used for all experiments. Five repeat analyses (Sun and Chen [2011]) of three independent set of matrix-free and matrix-matched calibration curves (at six levels of concentration of 2, 20, 50, 100, 200 and 400 μg mL^-1^) provided a total of 180 spectra.

### Materials and reagents

The matrix blanks applied in this study comprised of a complex mixture of Lingzhi fermentation solution and collagen. Within this mixture, the Lingzhi fermentation solution represents >95% by weight. Methanol (MeOH) and acetonitrile (MeCN) were of HPLC grade from Labscan. H_2_O was purified by passing through a Purelab Option-Q water purification system (Elga, UK). Raw materials of **(C)** and **(G)** were received in powdered form. Prior to use, individual stock solutions containing 100 mg mL^-1^ of **(C)** and **(G)** were prepared by extracting 10 g of each reference materials using 100 mL of MeOH for 10 min in a pear-shaped separating flask. The extracts were then transferred into 50 mL tapered tubes and centrifuged at 3, 226g for 6 min at 6°C. The supernatants were allowed to warm to room temperature before being purified by passing through a 0.2 μm PTFE syringe filter, and stored at 4°C in a 100 mL round bottom flask. From these individual stock solutions, mixed calibrants of both **(C)** and **(G)** were freshly prepared, by dilution with MeOH, at levels of 2, 20, 50, 100, 200 and 400 μg mL^-1^.

#### Statistics and data processing

##### Artificial neural network (ANN)

The mass spectra (counts versus mass/charge values for *m*/*z* 100 to 700) were exported to Excel 2007 (Microsoft Inc., Belleview, WA, USA) for data pre-processing and then to JMP 9.0 (division of SAS Institute Inc, Cary, North Carolina, USA) to perform artificial neural network (ANN) analysis (Onofrejová et al. [2007]; Korel and Balaban [2008]). ANN analysis was used to predict the amount of **(C)** and **(G)** in known samples by spiking. Briefly, the TanH activation function was applied using one hidden layer. Data obtained post-processing using Excel 2007 were fed into a hyperbolic tangent function (Belgrano et al. [2001]) represented by (*e*^*2x*^ - 1)/(*e*^*2x*^ + 1) that transforms values between −1 and 1. It is the centred and scaled version of the logistic function, with *x* representing the linear combination of the X variables. In this study, we assigned the intensities of the *m*/*z* fingerprints as the X variables, and concentrations as the Y variable. No penalty constraint (unsupervised) was applied to the model optimization step. To assess if the ANN model possess predictive capabilities, six independent spike recoveries at three levels of concentration (83, 166 and 333 mg kg^-1^) with five repeat analyses were performed.

#### Sample preparation

100 mL of acetonitrile was added to a 60 g aliquot of beverage in a pear-shaped extraction vessel and shake for 10 min. The mixture was transferred into 50 mL tapered tubes and centrifuged at 3, 226g for 6 min at 6°C. The supernatants were allowed to warm to room temperature before being purified by passing through a 0.2 μm PTFE syringe filter. 1 mL of the purified extract was transferred into a clear vial and injected (5 μL) into the LC-MS/MS for analysis.

#### Evaluation of signal enhancement/suppression effect and detection limit

A 60 g aliquot of Lingzhi fermentation solution and collagen was extracted as reported under “Sample preparation” and spiked with **(C)** and **(G)** at six levels of concentration (2, 20, 50, 100, 200 and 400 μg mL^-1^). Spiking levels (in milligrams per kilogram) were determined (Lim et al. [2011a]) as:

(1) $$[\left(\text{Concentration}of\;standard\;at\;each\;level\;of\;concentration\right)\times \left(\text{extraction }volume\right)\times 1]/\left(\text{weight }of\;matrix\right)$$

By applying this formula, the six levels of spiking concentration were determined as approximately 3, 33, 83, 166, 333 and 666 mg kg^-1^, respectively. For each concentration level, the signal enhancement or suppression effect was assessed as

(2) $$100–\left[\left(\text{predicted }concentration\left(C_{\text{pm}}\right)of\;matrix-matched\;standard\right)/\left(\text{predicted}concentration\left(C_{\text{ps}}\right)of\;matrix-free\;standard\right)\right]\times 100$$

where **C**_pm_ and **C**_ps_ were expressed as

(3) $$F\left(C_{\text{pm}},C_{\text{ps}}\right)=\{\begin{array}{l} \text{H1 }:\text{ TanH}\left[0.5*\sum \left({\text{a}}_{1}+{\text{ b}}_{1}*m/z_{1}+{\text{ c}}_{1}*m/z_{2}+{\text{ d}}_{1}*m/z_{3}+\ldots +{\text{ z}}_{1}*m/z_{\text{n}}\right)\right]\\ \text{H2 }:\text{ TanH}\left[0.5*\sum \left({\text{a}}_{2}+{\text{ b}}_{2}*m/z_{1}+{\text{ c}}_{2}*m/z_{2}+{\text{ d}}_{2}*m/z_{3}+\ldots +{\text{ z}}_{2}*m/z_{\text{n}}\right)\right]\\ \text{H3 }:\text{ TanH}\left[0.5*\sum \left({\text{a}}_{3}+{\text{ b}}_{3}*m/z_{1}+{\text{ c}}_{3}*m/z_{2}+{\text{ d}}_{3}*m/z_{3}+\ldots +{\text{ z}}_{3}*m/z_{\text{n}}\right)\right] \end{array}$$

The cross validated ANN model was defined by **F** (C_pm_,C_ps_) as three discreet TanH expressions, with constants (derived) and principal *m*/*z* values of **(C)** and **(G)** represented by (a_n_, b_n_, c_n_, …) and (*m*/*z*_n_), respectively. The final model output function is expressed as

(4) $$F\left(C_{\text{pm}},C_{\text{ps}}\right)=\alpha *\text{H}1+\beta *\text{H}2+µ*\text{H}3+\text{ constant}$$

where *α*, *β* and *μ* represent model specific constants.

In this study, it was necessary to utilize predicted concentration to calculate the signal enhancement/suppression effect instead of peak intensity as reported previously (Lim et al. [2011a]), as the models were trained to predict concentration as an outcome, not peak intensity.

Linearity was assessed using a residual plot. Limits of detection (LOD, *S*/*N* = 3) and limits of quantitation (LOQ, *S*/*N* = 10) were calculated basing on the signal-to-noise ratio for both matrix-free and matrix-matched **(C)** and **(G)** standards (relative to the solvent blank).

#### Recovery, intra-day and inter-day repeatability studies

An inter-day recovery study was conducted for two days. Six 5-g aliquots of blank Lingzhi fermentation solution and collagen were spiked with 50, 100 and 200 μg mL^-1^ (83, 166 and 333 mg kg^-1^) of **(C)** and **(G)** standards and extracted by performing a scaled-down protocol described under “Sample preparation” at a sample to solvent ratio of 3:5. Five repeat analyses per sample were performed using FI/MS and the **(C)** and **(G)** content determined by utilizing the matrix-matched **(C)** and **(G)** ANN models. It was necessary to construct separate ANN models for each analyte since the *m*/*z* values used to perform ANN analysis were different. Recovery (extraction efficiency) was assessed, expressed as [(mean *predicted concentration*)/(spiked *concentration*)] x 100.

## Results

### Mass spectra of *Cordyceps sinensis* and *Ganoderma lucidum*

**Figure 1:**
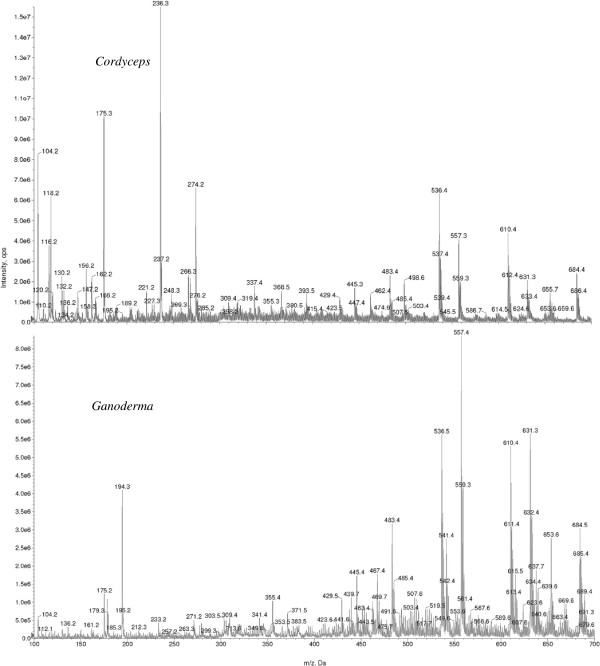
**FI/MS fingerprints of*Cordyceps*(top) and*Ganoderma*(bottom) at concentration level of 1 mg L**^**-1**^**obtained by performing EMS scan (positive mode) from*m*/*z*100 to 700 using the LIT.**

The enhanced MS scan spectra of **(C)** and **(G)** were characterized by feature-rich *m*/*z* values from *m*/*z* 100 to 700, as shown in Figure 1. The mass spectra of **(C)** and **(G)** collectively represent complex fingerprints of polysaccharide and adenosine amongst others (Lim et al. [2011b]). Owing to compositional differences of **(C)** and **(G)**, significant MS scan spectral variations were observed (P < 0.05), as shown in Figure 1(top and bottom), respectively.

### Artificial neural network analysis

The ANN model was constructed by applying a two-fold strategy namely model training and model validation, respectively. In model training, 90 spectra of each matrix-free and matrix-matched calibration curves were first trained using three hidden nodes. Based on the data, a cross validation was then performed by applying the K-fold cross-validation model. Briefly, K-fold method validation divided the original data into K subsets. In turn, each of the K sets was used to validate the model fit on the rest of the data, fitting a total of K models. The model that gave the best validation statistics was then chosen as the final model. The trained and cross validated models for **(C)** and **(G)** indicated that both matrix-free and matrix-matched calibration curves were linear over the relevant working range with the square of the correlation coefficients (*r*^2^) values between 0.9990 to 1.0000, as assessed from the individual residual plot (both training and validation models). Root-mean-square-deviation (RMSE) obtained for both **(C)** and **(G)** were highly satisfactory at <4% for both training and validation models.

### Signal enhancement /suppression effect in matrix

A summary of the signal enhancement/suppression effect on FI/MS detection using ANN analysis is listed in Table 1. From Table 1, the observed signal enhancement/suppression effect of **(C)** and **(G)** in Lingzhi fermentation solution and collagen ranged from −4% to 2% and −5% to 3%, respectively. These values were within the range accepted as highly satisfactory when multiple reaction monitoring (MRM) transition and MS^3^ were applied to perform analytical quantitation (Lim et al. [2011a]) using LIT.

**Table 1:** **Matrix effect on FI/MS detection of (C) and (G) in Lingzhi fermentation solution and collagen by applying FI/MS/ANN quantitation strategies at six (C) and (G) concentration levels (3, 33, 83, 166, 333 and 666 mg kg**^**-1**^**)**

**Concentration μg mL**^**-1**^**(mg kg**^**-1**^**)**	**(C)**	**(G)**
2 (3)	0	0
20 (33)	2	3
50 (83)	−4	−5
100 (166)	0	−1
200 (333)	2	2
400 (666)	−1	−2

The ion suppression (+) or enhancement (−) effect was assessed as 100 – [(predicted concentration (Cpm) of matrix-matched standard)/(predicted concentration (Cps) of matrix-free standard)] x 100. The values reported in the table have no unit.

### Evaluation of recovery, linearity, intra-day and inter-day repeatability

Six sets of Lingzhi fermentation solution and collagen blanks (n = 6) were spiked (83, 166 and 333 mg kg-1) prior to performing solvent extraction and five repeat analyses were performed per sample. This generated a total of 60 spectra. Recovery, intra-day and inter-day repeatability of FI/MS/ANN quantitation strategy were evaluated using matrix-matched standard calibration curves. The obtained LOQ, recoveries (mean) and relevant RSD (intra-day and inter-day) values are shown in Table 2, respectively, for **(C)** and **(G)** in Lingzhi fermentation solution and collagen. Matrix-matched calibration curves for both **(C)** and **(G)** were linear over the relevant working range with *r*^2^ values between 0.9994 and 0.9997, as assessed from the training model and cross-validated model outputs, respectively. A good recovery range of 92-118% was achieved for both **(C)** and **(G)** in matrix, as summarized in Table 2. Intra-day and inter-day RMSE values of 7% or better was achieved basing on the results obtained by applying FI/MS/ANN quantitation strategies.

**Table 2:** **Mean recoveries (in percent), RSD (intra-day and inter-day,*n*** **= 6) for matrix-matched (C) and (G) at three levels of spiking (83, 166 and 333 mg kg**^**-1**^**) measured over 2 days. Limits of quantitation (LOQ, S/N = 10) for matrix matched (C) and (G) expressed as milligrams per liter and milligrams per kilogram**

**Analytes**	**Mean Recoveries**^**a**^**(%, RSD**_**intra-day**_**, RSD**_**inter-day**_^**b**^**)**			**LOQ**	
	**83 mg kg**^**-1**^	**166 mg kg**^**-1**^	**333 mg kg**^**-1**^	**mg L**^**-1**^	**mg kg**^**-1**^
(C)	92 (4, 7)	103 (3, 4)	115 (2, 4)	0.2	0.3
(G)	105 (3, 6)	100 (4, 4)	116 (2, 3)	0.2	0.3

^a^ Recovery percentages were calculated using matrix-matched calibration curves.

^b^ RSD values for intra-day and inter-day were shown in parentheses, respectively.

## Discussion

The objective of this study was to quantify the amount of **(C)** and **(G)** present in a beverage containing complex ingredients of Lingzhi fermentation solution and collagen in minutes without performing chromatographic separation. As a first step, the MS fingerprints for **(C)** and **(G)** were obtained as the sum of all the spectra between 0 and 1.0 min, at six levels of concentration (2, 20, 50, 100, 200 and 400 μg mL^-1^). Next, *m*/*z* values that were specific to **(C)** and **(G)** were identified.

The process of identifying meaningful *m*/*z* values principal to **(C)** and **(G)** was non-trivial since conventional strategy involved the application of mathematical procedures that transformed a number of possibly correlated variables into smaller number of uncorrelated variables by applying principal component analysis (PCA). Indeed, Sun and Chen ([2011]) reported the application of PCA and analysis of variance (ANOVA) to authenticate *Scutellaria lateriflora* in dietary supplements. In their study, *m*/*z* values specific to *Scutellaria lateriflora* were known. In this study, however, the *m*/*z* values salient to both **(C)** and **(G)** were not known. By applying PCA and ANOVA analyses, we found that such dimension reduction strategy did not contribute toward the successful identification of meaningful *m*/*z* values that were principal to **(C)** and **(G)**. One possible reason could be the data exhibited high variance with a paucity of replicates, thus providing a steep challenge to data mining (Enot et al. [2008]). Indeed, if we refer to Figure 1 showing the EMS spectra of both **(C)** and **(G)**, the former MS spectra appear heavily weighted in the lower *m*/*z* range from 100 to 500, as compared to the latter that is weighted from *m*/*z* range 400 to 700. Simply, by applying a combination of PCA and ANOVA analyses without taking into account the weights of the relevant *m*/*z* ranges of individual spectra of **(C)** and **(G)**, we run the risk of identifying false *m*/*z* values, thereby providing non-representative snapshots of the chemical content of the individual samples of **(C)** and **(G)**.

To address this limitation, we applied a modified spectroscopic quantitation workflow reported previously (Lim et al. [2011a]). Briefly the principal *m*/*z* values of **(C)** and **(G)** were identified by considering the correlation coefficient criteria of > = 0.995 by plotting the normalized intensity (Meuleman et al. [2008]; Deininger et al. [2011]) of the *m*/*z* values versus concentrations. Normalization was performed by utilizing Analyst 1.5.0 (AB SCIEX, Foster city, CA, USA) for windows. Within the framework of full MS spectra pattern recognition and its application to distinguish genera and species (Chen et al. [2010]; Harnly et al. [2009]), the principal *m*/*z* values of **(C)** and **(G)** were identified as: 104.2, 116.2, 120.2, 175.2, 236.3, 248.3, 266.3, 366.6 and 498.6; 439.7, 469.7, 511.7, 551.6, 623.6, 637.7 and 653.6, respectively. By utilizing multiple *m*/*z* values specific to **(C)** and **(G)**, undue reliance on one or two marker *m*/*z* values was lifted, thereby increasing the robustness of the analysis.

Indeed, albeit the concept of utilizing correlation coefficient criteria as a strategy to identify prominent principal **(C)** and **(G)***m*/*z* values is relatively new to the framework of FI/MS, it is well-adapted in the field of vibrational spectroscopy (Lim et al. [2011a]). In vibrational spectroscopy, absorption band characteristic of the standard materials were first identified and (their suitability) assessed by performing spike recovery studies in matrix. The results obtained were then used to construct a statistical model to predict concentrations of control samples. To construct a statistical model that possessed predictive capability, it was essential that absorption bands of the analyte and those of the matrix do not overlap: spectral overlapping gave rise to spectral interference, thereby rendering the results unusable for further statistical analysis. Within the framework of mass spectrometry, however, the limitation associated with mass spectra overlap in spectroscopy is lifted in that single *m*/*z* value does not relate to individual analyte present in the herbal extracts; chemical fingerprints from different analytes can contribute to several *m*/*z* signals in the spectra and it is equally common for several analytes to contribute to the same *m*/*z* value (Enot et al. [2006]). By the same strategy but applied to the framework of FI/MS, in order to assess if the identified principal *m*/*z* values were useful for predicting concentrations of control samples accurately through spiking experiments, a statistical model (ANN) was constructed by utilizing intensities of individual *m*/*z* values of **(C)** and **(G)** at their respective concentration levels (2, 20, 50, 100, 200 and 400 μg mL^-1^).

While the models obtained for both **(C)** and **(G)** appeared well trained and validated, we found that it was necessary to introduce some noise into the respective models to assess if the models were sensitive to statistical outlier due to repeat analyses using MS, as judged basing on the RMSE values. The inclusion of this additional step was to address concerns associated with possible *m*/*z* signal confusion (with instrumental artifacts) discussed by Enot and colleagues for experiments involving no chromatographic step. To achieve the objective of enhanced noise to signal data separation, spectra of solvent blanks (representing noise) comprising 50:50 (v/v) mobile phases of 0.1% formic acid (A) and MeOH (B) were entered into the ANN models, where the process of model training and cross validation was reiterated by utilizing the same K-fold condition and three hidden nodes. As expected, the RMSE and *r*^2^ values for both **(C)** and **(G)** basing on the results obtained by applying the individual cross validated model changed from <4% to >10%, and from 0.9990 to 0.8900, respectively. The increase in RMSE values and reduction in *r*^2^ values suggested that the cross validated models for **(C)** and **(G)**, when fully optimized, were sufficiently robust in identifying noise in the data. The high sensitivity of the RMSE and *r*^2^ values with respect to noise in the data highlighted the need to perform an additional data pre-processing step prior to performing statistical analyses. Indeed, our observation is in good agreement with protocols reported by other researchers (Enot et al [2006]; Broadhurst and Kell [2006]). One possible solution to achieve data integrity would be to consider applying t-test as a data pre-processing step. In this study, however, no data cleanup was performed, thereby suggesting that the LIT was robust as a tool when applied to perform repeat analyses.

With the integrity of both models ascertained, efforts were then focused on studying the signal enhancement/suppression effect in matrix.

In order to better understand signal enhancement/suppression due to interfering ions and distinguish such effect from poor recovery due to solvent extraction efficiency, spiking experiments were performed at six levels of concentration (2, 20, 50, 100, 200 and 400 μg mL^-1^) post solvent extraction step. Carry over was initially detected in the solvent blank after each sample injection. For this reason, FI/MS experiments were performed by adding an equilibrium step of 2 min between injections to allow the residual *m*/*z* values to be purged from the LC system completely. As the salient *m*/*z* values of **(C)** and **(G)** were unique, the absence of these salient *m*/*z* values in the MS spectra was used as an indicator of a good solvent blank.

By referring to LOQ values shown in Table 2, two observations were made: the LOQ values for **(C)** and **(G)** were both pinned; the LOQ values were significantly higher than the detection limit of the QTrap 5500 MS instrument, reported previously (Lim et al. [2011a]). Indeed, owing to the direct flow injection strategy applied in the entirety of the method validation, we observed severe precipitation (technical difficulties) occurring on the cone surface. This enhanced precipitation contributed to severe random arcing at the source tip, thereby rendering data (integrity) obtained at lower concentration levels to be compromised. For this reason, the ANN models constructed using these compromised data only achieve *r*^2^ values of 0.7 or poorer at high RMSE values (>50%). To achieve a fine balance between the integrity of the LIT (protect from contamination due to direct flow injection) and reasonable method sensitivity, the curtain gas value used to perform MS analyses was therefore increased from 25 to 30. This increase in curtain gas value (from 25 to 30) gave rise to a resultant 10 times reduction in method sensitivity, which explained the LOQ values pinning phenomena observed for both **(C)** and **(G)**. Therefore, the LOQ values reported in Table 2 should be interpreted as a conservative representation of the method true capability.

Indeed, while the application of this adapted scaffold may be suitable for other bacteria analysis as well, it is equally important that synthetically cultivated bacteria and naturally occurring bacteria are differentiated since the former would possibly contain a relatively more replicable MS fingerprint akin to those of pharmaceutical produce. For this reason, the implementation of this adapted scaffold in a high-throughput production plant would imply a need to perform materials reassessment to suitably address ANN model integrity concerns associated with batch to batch materials variations.

In summary, an accurate, highly selective and reliable method utilizing FI/MS/ANN quantitation strategy of **(C)** and **(G)** in complex matrix containing Lingzhi fermentation solution and collagen was developed. The application of a simplified rule-based workflow (*r* > =0.995) via ANN analyses facilitated an easy, accurate and fit-for-purpose solution toward the identification of features salient to extracts of **(C)** and **(G)** without the need to perform chromatographic separation. The high throughput capability offered by applying direct flow injection strategy, together with the enhanced selectivity and robustness enabled by utilizing multiple *m*/*z* values distilled via ANN modeling pathways, are perhaps resolutions to fields that demand analyte specificity and MS fingerprinting quantitation capabilities that are not easily achievable when multiple reaction monitoring (MRM) transition and MS^3^ are applied. Application of this adapted scaffold in a high-throughput routine environment (such as herbal products manufacturing plant) would imply a significant reduction in effort and time, since the option of having a model driven analytical solution is now available.

## Competing interests

The authors declare that they have no competing interests.

## References

[B1] Beckmann M Parker D Enot DP Duval E Draper J High-throughput, nontargeted metabolite fingerprinting using nominal mass flow injection electrospray mass spectrometry Nat Protoc 2008 3 486 504 18323818 10.1038/nprot.2007.500

[B2] Belgrano A Malmgren BA Lindahl O Application of artificial neural networks (ANN) to primary production time-series data J Plankton Res 2001 23 651 658 10.1093/plankt/23.6.651

[B3] Boto VI Sakkas VA Albanis TA An experimental design approach employing artificial neural networks for the determination of potential endocrine disruptors in food using matrix solid-phase dispersion J Chromatogr A 2009 1216 1296 1304 10.1016/j.chroma.2008.12.070 19144345

[B4] Broadhurst DI Kell DB Statistical strategies for avoiding false discoveries in metabolomics and related experiments Metabolomics 2006 2 171 196

[B5] Chen P Lin LZ Harnly JM Mass spectroscopic fingerprinting method for differentiation between Scutellaria lateriflora and the Germander (Teucrium canadense and T. chamaedrys) species J AOAC Int 2010 4 1148 1154 20922946 PMC3762689

[B6] Debska B Guzowska-Świder B Application of artificial neural network in food classification Anal Chim Acta 2011 705 283 291 10.1016/j.aca.2011.06.033 21962371

[B7] Deininger SO Cornett DS Paape R Becker M Pineau C Rauser S Walch A Wolski E Normalization in MALDI-TOF imaging datasets of proteins: practical considerations Anal Bioanal Chem 2011 401 167 181 10.1007/s00216-011-4929-z 21479971 PMC3124646

[B8] Enot DP Beckmann M Overy D Draper J Predicting interpretability of metabolome models based on behavior, putative identity, and biological relevance of explanatory signals Proc. Natl. Acad. Sci. USA 2006 103 14865 14870 10.1073/pnas.0605152103 16990432 PMC1595442

[B9] Enot DP Lin W Beckmann M Parker D Overy DP Draper J Preprocessing, classification modeling and feature selection using flow injection electrospray mass spectrometry metabolite fingerprint data Nat Protoc 2008 3 446 470 10.1038/nprot.2007.511 18323816

[B10] Galdón BR Peña-Méndez E Havel J Rodríguez EMR Romero CD Cluster analysis and artificial neural networks multivariate classification of onion varieties J Agric Food Chem 2010 58 11435 11440 10.1021/jf102014j 20949919

[B11] Harnly JM Pastor-Corrales MA Luthria DL Variance in the chemical composition of dry deans determined from UV spectral fingerprints J Agric Food Chem 2009 57 8705 8710 10.1021/jf900852y 19731933

[B12] Korel F Balaban MÖ Electronic nose technology in food analysis Handbook of Food Analysis Instruments 2008 10.1201/9781420045673.ch16

[B13] Kristiansen GK Brock R Bojesen G Comparison of flow injection/thermospray MS/MS and LC/thermospray MS/MS methods for determination of sulfonamides in meat and blood Anal Chem 1994 66 3253 3258 10.1021/ac00091a040 7978309

[B14] Lim CW Tai SH Lee LM Chan SH Analytical method for the accurate determination of tricothecenes in grains using LC-MS/MS: a comparison between MRM transition and MS3 quantitation Anal Bioanal Chem 2011 10.1007/s00216-011-5558-2 22209956

[B15] Lim CW Chan SH Visconti A Feed-forward neural network assisted by discriminant analysis for the spectroscopic discriminantion of cracked spores Ganoderma lucidum: A prospective biotechnology production tool AMB Express 2011 10.1186/2191-0855-1-40 PMC3240128 22082074

[B16] Lisec J Schauer N Kopka J Willmitzer L Fernie AR Gas chromatography spectrometry-based metabolite profiling in plants Nat Protoc 2006 1 387 396 10.1038/nprot.2006.59 17406261

[B17] Marini F Artificial neural networks in foodstuff analyses: Trends and perspectives. A review Anal Chim Acta 2009 635 121 131 10.1016/j.aca.2009.01.009 19216869

[B18] Meuleman W Engwegen JY Gast MC Beijnen JH Reinders MJ Wessels LF Comparison of normalization methods for surface-enhanced laser desorption and ionization (SELDI) time-of-flight (TOF) mass spectrometry data BMC Bioinformatics 2008 10.1186/1471-2105-9-88 PMC2258289 18257918

[B19] Mol HGJ Plaza-Bolaños P Zomer P de Rijk TC Stolker AAM Mulder PPJ Toward generic extraction method for simultaneous determination of pesticides, mycotoxins, plant toxins, and veterinary drugs in feed and food matrices Anal Chem 2008 80 9450 9459 10.1021/ac801557f 19072261

[B20] Nanita SC Pentz AM Bramble FQ High-throughput pesticide residue quantitative analysis achieved by tandem mass spectrometry with automated flow injection Anal chem 2009 81 3134 3142 10.1021/ac900226w 19296591

[B21] Nanita SC High-throughput chemical residue analysis by fast extraction and dilution flow injection mass spectrometry Analyst 2011 136 285 287 10.1039/c0an00720j 21046028

[B22] Nanita SC Stry JJ Pentz AM McClory JP May JH Fast extraction and dilution flow injection mass spectrometry method for quantitative chemical residue screening in food J Agric Food Chem 2011 59 7557 7568 10.1021/jf104237y 21388127

[B23] Onofrejová L Farková M Preisler J Quantitative MALDI MS analysis of food oligosaccharides using artificial neural networks7th International Conference Vitamins, Nutrition and Diagnostics 2007 18 20

[B24] Roddy TP Horvath CR Stout SJ Kenney KL Ho P-I Zhang J-H Vickers C Kaushik V Hubbard B Wang YK Anal Chem 2007 79 8207 8213 10.1021/ac062421q 17902631

[B25] Sun J Chen P A flow-injection mass spectrometry fingerprinting method for authentication and quality assessment of Scutellaria lateriflora-based dietary supplements Anal Bioanal Chem 2011 401 1581 1588 10.1007/s00216-011-5246-2 21773734

[B26] Ward JL Harris C Lewis J Beale MH Assessment of H-1MNR spectroscopy and multivariate analysis as a technique for metabolite fingerprinting of Arabidopsis thaliana Phytochemistry 2003 62 949 957 10.1016/S0031-9422(02)00705-7 12590122

[B27] Yang FQ Ge L Yong JWH Tan SN Li SP Determination of nucleosides and nucleobases in different species of Cordyceps by capillary electrophoresis–mass spectrometry J Pharm Biomed Anal 2009 50 307 314 10.1016/j.jpba.2009.04.027 19497699

